# Artificial intelligence: The researcher's assistant or sheep in wolf's clothing?

**DOI:** 10.1002/ueg2.12689

**Published:** 2024-11-07

**Authors:** Pisani Anthea, Campani Claudia

**Affiliations:** ^1^ Division of Gastroenterology Department of Medicine Mater Dei Hospital Msida Malta; ^2^ Internal Medicine and Liver Unit University Hospital Careggi University of Florence Florence Italy; ^3^ Department of Experimental and Clinical Medicine University of Florence Florence Italy

Artificial intelligence (AI) has taken the world by storm; from virtual assistants to cybersecurity, from navigation apps to automated vehicles, it has become an integral part of our everyday lives. The medical field has also employed AI in various domains, including, amongst others, oncology, where it is utilised to manage large quantities of data simultaneously or during endoscopy to aid in the detection of premalignant lesions. It is no surprise, therefore, that AI has been applied in medical research and in the publishing field. This phenomenon raises a lot of questions: Am I allowed to use AI when writing a research paper and, if yes, to what extent? Will AI increase or decrease the chances of my paper being published?

## HOW CAN AI BE USED IN RESEARCH PAPER WRITING?

There are several tools available from the AI armamentarium to support in research paper‐writing. Writing assistance models can provide paraphrasing of a supplied text or give suggestions to correct grammatical mistakes and improve the quality of the text whilst translation services can convert texts from the user's native language to another, usually English. These tools can potentially level the playing field for researchers struggling with English, especially in light of studies showing that non‐native English speakers spend more effort than their English‐speaking counterparts in writing their research papers.^1^ Additionally, there are allegations of linguistic injustice suggesting that manuscripts that do not confirm to perceived standards of international academic English might be more likely to be rejected.^2^, ^3^, ^4^

With content‐generation models, different platforms are able to generate text based on the prompts given by the user. By far one of most commonly used, and discussed, platforms is ChatGPT, which was introduced in November 2022 by OpenAI. The chatbot uses algorithms designed to understand the input by the user and provide either pre‐written or AI‐generated responses and is continually being optimised by reinforcement learning from human feedback.^5^, ^6^ The free version of ChatGPT offers basic capabilities suitable for general data analytics inquiries, providing users with standard responses and a limited understanding of complex data sets. In contrast, the paid version provides enhanced performance, including faster response times and improved accuracy. Users of the paid version can upload large excel files and request specific analyses and a result output or assistance in generating Phyton or R codes to analyse their data. In addition, the platform can be asked to generate images based on their data or a specific text.

AI research assistants, such as typeset.io and elicit.org are platforms that incorporate various tools to assist authors in generating and formatting their research papers by performing literature reviews, providing explanations for academic texts, extracting data, arranging citations and paraphrasing. Such platforms can help researchers gain an overview of a specific topic.

Since the introduction of such models, several authors have explored the options of writing and publishing manuscripts with content generation models. These include case reports,^7^ editorials,^8^ a hypothetical research article^9^ and full articles.^10^ A recent systematic review summarized how 51.7% of the analysed manuscripts cited benefits with utilizing ChatGPT in the context of academic writing, mainly as a result of increasing efficiency, improving language and accelerating literature review while 33.3% of papers highlighted benefits in scientific research, especially with data analysis.^11^

## SHOULD AI BE USED IN RESEARCH PAPER WRITING?

Using AI for research analysis and presentation can be tempting, but researchers must be aware its drawbacks and potential pitfalls.

### Data inaccuracies and lack of critical analysis

Several authors have experimented with the use of AI in various steps of the publishing process. Amongst others, Manohar and Prasad^7^ used ChatGPT to generate content for their case report on seronegative systemic lupus erythematosus in a patient with HIV infection. They found that the AI‐generated text contained inaccurate scientific information and non‐existing citations. Concerns on the risk of incorrect data is frequently mentioned since AI lacks human expertise and critical thinking therefore it can draw incorrect conclusions, thereby resulting in the potential to spread misinformation. Therefore, any AI‐generated material should be manually checked by an expert in the field. The occurrence of incorrect references is not new,^9^, ^12^ with the term “data hallucination” being coined to describe seemingly invented references and data. Athaluri et al^12^ devised an experiment to investigate the references generated by ChatGPT and found that out of the 178 references generated by ChatGPT, 69 lacked a valid DOI (digital objective identifier) and 28 could not be found.

AI‐generated research summaries should not be taken at face value. These summaries can be generic and lack critical analysis, drawing “easy” conclusions without properly analysing the strengths and limitations of specific studies. This issue is compounded if only abstracts are used, particularly for non‐open access articles.

Data analysis by AI platforms is enticing, but to use the results effectively, researcher need a strong foundation in data analytics and must provide precise and detailed requests regarding the type of analysis or images desired.

### Ethical considerations

Misuse of AI can lead to fraudulency in research, such as ghost‐writing and fabricated data.^13^, ^14^ Unfortunately, misconduct in research paper‐writing is present, with a systematic review reporting that 2% of researchers admitting to fabricating, falsifying and modifying their data.^15^

Detecting AI‐generated text is challenging despite reliable AI‐checker tools and Editors often rely on intuition. Indicators of AI writing include repetitive phrases, certain buzzwords,^16^ vague yet lengthy descriptions, itemized text and a lack of fluidity or cohesion. Whilst AI‐detection apps can give an indication as to whether material is AI‐generated, their effectiveness remains questionable^17^ and their application raises ethical questions: is the use of AI justified for detecting AI? Identifying AI‐generated content is crucial for maintaining the integrity of academic and professional work, which requires knowledge, critical thought and creativity.

The use of AI in research paper writing can lead to issues regarding authorship and accountability. Interestingly, two articles initially listed ChatGPT as an author but both later moved it to acknowledgement section, as Large Language Models (LLM) such as ChatGPT do not currently meet authorship criteria and cannot be held accountable.^18^, ^19^ Publishing houses, as well as Academic Associations and Committees including the International Committee of Medical Journal Editors (ICMJE) and the Committee on Publication Ethics (COPE) have issued statements claiming that AI platforms cannot be listed as authors.

Another important aspect to consider would be data confidentiality and privacy issues, especially when patient data is uploaded. In view of the risk of unauthorised access, data breaches and possible patient reidentification, participants in a study need to be informed, and consented, for their data to be utilised by an AI platform.

Plagiarism is another concern when using AI. Aydin et al^20^ employed ChatGPT to both paraphrase abstract texts and to generate a literature review based on prompts by the authors. They then used a plagiarism tool to assess the matching rate and compared that with text written by authors, noting that the matching rate was 40% for the paraphrasing tool. This finding, however, was not reproduced in a study by Altmäe et al,^9^ who found a similarity index of only 19%. Some AI models are specifically designed to paraphrase to avoid plagiarism detection, but ethical questions remain about rewording another author's work, without any critical appraisal or personal input.

Various publishing houses, including Wiley, Cambridge University Press, Elsevier and Springer Nature currently require that authors declare and explain whether and how AI has been used in the research and preparation of the paper.

## CONCLUSION

When all of this is put together, AI has the potential to assist researchers in performing their research and writing their research papers, however it should be used with caution. AI platforms can expedite tasks and provide a general overview of a topic; however, the product should not be applied directly but needs to be critically analysed by the researcher to assess whether the interpretation is medically sound, the citations are the most relevant ones and the conclusions have a strong medical basis. Whilst future updates and training can fix some of AI's current weaknesses, AI cannot replace the inherently human traits of the researcher.

Furthermore, as researchers we owe it to each other, and to the readers, to uphold the ethical standards associated with research and writing. This is even more imperative when clinical implications that can impact patient management are made.

Finally, while these advanced features offer significant convenience, one might also ponder whether it is truly worthwhile to limit learning and effort, or if the joy lies in the whole process that results not only in one's original work, but also in the personal growth of the researcher.

## CONFLICT OF INTEREST STATEMENT

The authors do not have any conflicts of interest to disclose.

## Data Availability

Data sharing not applicable to this article as no datasets were generated or analysed during the current study.
